# *Candida albicans* Dispersed Cells Are Developmentally Distinct from Biofilm and Planktonic Cells

**DOI:** 10.1128/mBio.01338-18

**Published:** 2018-08-21

**Authors:** Priya Uppuluri, Maikel Acosta Zaldívar, Matthew Z. Anderson, Matthew J. Dunn, Judith Berman, Jose Luis Lopez Ribot, Julia R. Köhler

**Affiliations:** a The Division of Infectious Diseases, Los Angeles Biomedical Research Institute at Harbor, University of California Los Angeles (UCLA) Medical Center, Los Angeles, California, USA; b Division of Infectious Diseases, Children’s Hospital, Boston, Massachusetts, USA; c Department of Microbiology, the Ohio State University, Columbus, Ohio, USA; d Department of Molecular Microbiology & Biotechnology, Tel Aviv University, Ramat Aviv, Israel; e Department of Biology and South Texas Center for Emerging Infectious Diseases, the University of Texas at San Antonio, San Antonio, Texas, USA; Washington University School of Medicine

**Keywords:** *Candida albicans*, RNA-seq, biofilms, carbon metabolism, dispersal, gene expression, planktonic

## Abstract

Candida albicans surface-attached biofilms such as those formed on intravenous catheters with direct access to the bloodstream often serve as a nidus for continuous release of cells capable of initiating new infectious foci. We previously reported that cells dispersed from a biofilm are yeast cells that originate from the top-most hyphal layers of the biofilm. Compared to their planktonic counterparts, these biofilm dispersal yeast cells displayed enhanced virulence-associated characteristics and drug resistance. However, little is known about their molecular properties. To address that issue, in this study we aimed to define the molecular characteristics of these biofilm dispersal cells. We found that the inducer of dispersal, *PES1*, genetically interacts with the repressor of filamentation, *NRG1*, in a manner consistent with the definition of dispersed cells as yeast cells. Further, using a flow biofilm model, we performed comprehensive comparative RNA sequencing on freshly dispersed cells in order to identify unique transcriptomic characteristics. Gene expression analysis demonstrated that dispersed cells largely inherit a biofilm-like mRNA profile. Strikingly, however, dispersed cells seemed transcriptionally reprogrammed to acquire nutrients such as zinc and amino acids and to metabolize alternative carbon sources, while their biofilm-associated parent cells did not induce the same high-affinity transporters or express gluconeogenetic genes, despite exposure to the same nutritional signals. Collectively, the findings from this study characterize cell dispersal as an intrinsic step of biofilm development which generates propagules more adept at colonizing distant host sites. This developmental step anticipates the need for virulence-associated gene expression before the cells experience the associated external signals.

## IMPORTANCE

Candida albicans surface-attached biofilms serve as a reservoir of cells to perpetuate and expand an infection; cells released from biofilms on catheters have direct access to the bloodstream. Biofilm dispersal yeast cells exhibit enhanced adhesion, invasion, and biofilm formation compared to their planktonic counterparts. Here, we show using transcriptome sequencing (RNA-seq) that dispersed yeast cells are developmentally distinct from the cells in their parent biofilms as well as from planktonic yeast cells. Dispersal cells possess an anticipatory expression pattern that primes them to infect new sites in the host, to survive in nutrient-starved niches, and to invade new sites. These studies identified dispersal cells as a unique proliferative cell type of the biofilm and showed that they could serve as targets for antibiofilm drug development in the future.

## INTRODUCTION

Detachment of microorganisms from an established site of proliferation mediates spread of pathogens to new sites or through the bloodstream, resulting in disseminated disease. Frequently, a pathogen’s initial proliferative nidus consists of its presence on a biofilm, on a mucosal or mesothelial surface, or on a foreign body.

The human commensal Candida albicans is the most frequently isolated human fungal opportunistic pathogen. Disseminated candidiasis carries high mortality rates despite appropriate antifungal drug treatment (1, 2). C. albicans is unique in its ability to switch between growth forms in the host, i.e., between budding yeast and filamentous pseudohyphae and hyphae. Morphological switching enhances its ability to adhere and invade and to sustain a community of biofilm cells. Decades of research have elucidated the regulation of hyphal morphogenesis and its association with pathogenesis (3–5). Most recently, candidalysin, a toxin secreted by C. albicans, was shown to be produced only during hyphal growth (6).

Hyphal filaments constitutively produce cells exhibiting lateral compartmentation (lateral yeast cells) on their subapical segments, while apical segments continue to extend as filamentous cells. Lateral yeast cells are formed *in vivo*, since filamentous cells are typically seen in conjunction with yeast cells in organs of hosts with invasive candidiasis (7, 8). C. albicans biofilms are initiated when yeast cells adhere to a surface and form microcolonies. Over time, the cells differentiate into hyphae that eventually develop into a complex community of basal yeast cells and into layers of hyphal cells encased in a blanket of self-produced polysaccharide matrix (9, 10). Throughout the biofilm growth cycle, hyphae continuously release lateral yeast cells (10). This phenomenon is of great clinical relevance, as cells released from a biofilm formed on an indwelling catheter or an infectious nidus can gain access to the bloodstream, disseminate, and initiate distant foci of infection. Although our current understanding of lateral yeast production and biofilm dispersal is limited, we have previously demonstrated that yeast cells arising from biofilms adhere better to mammalian cells, are more resistant to azole drugs, and display higher virulence in a disseminated mouse model of candidiasis (10). Thus, dispersal of cells from biofilms appears to be a distinct developmental phase of the fungus in which cells are primed for invasion of the host.

To date, Pes1 is the only molecular regulator that has been shown to control production of lateral yeast cells from hyphae and to induce biofilm dispersal (10–12). Pes1 regulates lateral yeast cell production but not hyphal morphology or biofilm architecture and has been shown to be essential for sustained candidiasis (7). In fact, our previous studies demonstrated that curtailing lateral yeast cell release from tissue-invading hyphae by depleting *PES1* significantly reduced dissemination and virulence in mice (8). In light of these findings, the current study was designed to elucidate the molecular characteristics of lateral yeast cells released from biofilms.

Here, we used RNA sequencing to delineate biofilm dispersal cell-specific molecular signatures and to identify gene expression patterns that might set the dispersed cells apart from their parent biofilms or their planktonic counterparts. We found that the transcriptional landscape of dispersed yeast largely overlapped that of their parent hyphal biofilms, rather than that of the morphologically similar planktonic yeast cells. Virulence-associated genes were a substantial component of the upregulated set. Despite arising within the same nutritional milieu, dispersed cells and biofilm cells exhibited striking contrasts in their metabolic gene expression profiles which suggested an anticipatory developmental step that takes place during dispersal cell formation, during which cells are prepared for an invasive role before they are released from the biofilm. In a jugular vein-catheterized mouse model, we showed that dispersed yeast cells are the link between biofilm-infected catheters and the foci of infection in a deep organ. In summary, we defined molecular characteristics of biofilm dispersal cells and show that they are in a developmental phase distinct from the biofilm or planktonic state and are specifically equipped for immediate infection in the host.

## RESULTS

### Transcript profiles of biofilm dispersal cell populations strongly corresponded to those of biofilm cells.

To understand the molecular characteristics of cells dispersed from biofilms, we collected and characterized yeast cells spontaneously released from C. albicans biofilms, using the simple flow biofilm model (13, 14). Age-matched planktonic and biofilm cells were also recovered from biofilms grown for 24 h, and transcript profiles of the 3 cell populations were delineated using transcriptome sequencing (RNA sequencing [RNA-seq]).

**FIG 1:**
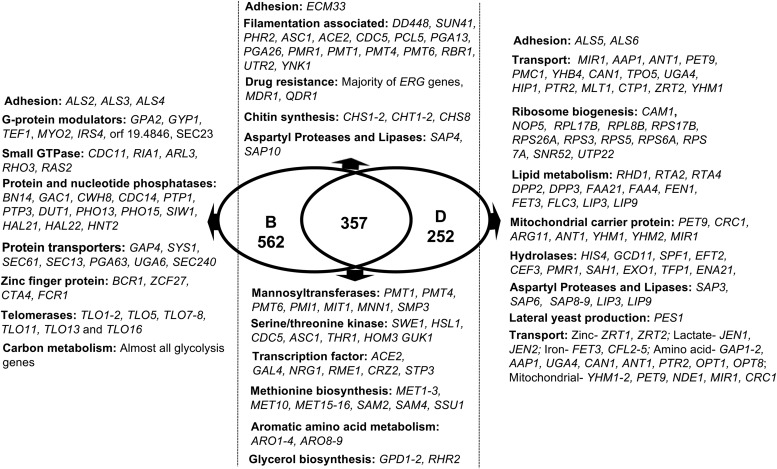
Venn diagram of significantly upregulated genes in biofilms and biofilm dispersal cells at 24 h, compared to planktonic cells. Gene expression profiles of C. albicans biofilm (B) and biofilm dispersal cells (D) were each compared individually to age-matched planktonic cells (P). The Venn diagram displays the total number of genes upregulated under each growth condition and those common between biofilms and dispersed cells. The diagram also shows some genes and their functional categories (additional to those mentioned in the body of the manuscript) whose levels are elevated in each group.

To validate our approach, we first compared the expression patterns of biofilm and planktonic cells. Results from the analysis showed a total of 1,524 genes to be significantly altered in expression (using a false-discovery rate [adjusted *P* value {p-adj}] of <0.05) between the 2 cell types. Our comparison of biofilm cells and free-living cells largely replicated microarray-based studies by others (15, 16), concurrently validating our procedure (Fig. 1; see also Data Set S1 in the supplemental material).

10.1128/mBio.01338-18.2 DATA SET S1  Comprehensive differentially expressed data of significantly (*P* < 0.5) regulated (>1.5-fold) genes. In this Excel sheet, sheet 1 lists genes significantly upregulated in biofilm versus planktonic cells, sheet 2 lists genes significantly downregulated in biofilm versus planktonic cells, sheet 3 lists genes common between our study and other transcriptone studies comparing biofilm expression data to planktonic expression data, sheet 4 lists genes significantly upregulated in dispersed versus planktonic cells, sheet 5 lists genes significantly downregulated in dispersed versus planktonic cells, sheet 6 lists genes showing similar patterns of upregulation in both biofilm and planktonic cells compared to planktonic cells, and sheet 7 lists genes showing similar patterns of downregulation in both biofilm and planktonic cells compared to planktonic cells. Download DATA SET S1, XLSX file, 0.3 MB. Copyright © 2018 Uppuluri et al. 2018 Uppuluri et al. This content is distributed under the terms of the Creative Commons Attribution 4.0 International license.

Next, we compared the biofilm dispersal cells to their age-matched planktonic and biofilm sisters. A total of 963 genes showed statistically significant differences between the dispersed and planktonic conditions (*P* < 0.05). Of the 609 genes upregulated >1.6-fold in dispersed cells, 357 (~59%) were also upregulated in cells from a mature biofilm compared to planktonic cells (Data Set S1). Regulatory patterns of dispersed cells substantially reflected those of their parent biofilms with respect to genes involved in methionine biosynthesis (*MET1*, *MET2*, *MET3*, *MET10*, *MET15*, *MET16*, *SAM2*, *SAM4*, and *SSU1*) (15), aromatic amino acid metabolism (*ARO2*, *ARO3*, *ARO4*, *ARO8*, and *ARO9*), ergosterol biosynthesis (a majority of *ERG* genes), adhesion (*ECM33*) (17, 18), small-molecule efflux and drug resistance (*MDR1* and *QDR1*) (19, 20), chitin synthesis (*CHS1*, *CHS2*, *CHS8*, *CHT1*, and *CHT2*) (21), and glycerol biosynthesis (*GPD1*, *GPD2*, and *RHR2*) (22); almost all ribosome biogenesis genes (15, 23); and many others defined previously as biofilm regulated (24). Expression of the yeast-specific *YWP1* gene (25) was 2-fold higher in dispersed cells than in biofilms and was higher in both than in planktonic cells.

Similarly, the majority of the *SAP* family genes, *SAP3*, *SAP6*, *SAP8*, and *SAP9*, were found to be upregulated in dispersed cells, while the expression levels of *SAP4* and *SAP10* were also elevated in biofilm cells. Similarly, *LIP3* and *LIP9* were upregulated in dispersed cells, with *LIP4* and *LIP6* expression levels elevated only in biofilm cells. None of the SAP or lipase family genes were expressed in planktonic cells, similarly to previous reports (26).

### Dispersed yeast cells expressed genes associated with the hyphal form.

Despite their yeast morphology and the expression of two predominantly yeast-specific genes (*YWP1* and *RHD3*) (27), several genes typically expressed in hyphae rather than in yeast cells, including *DDR48*, *PHR2*, *ASC1*, *SUN41*, *ACE2*, *CDC5*, *CHA1*, *PCL5*, *PGA13*, *PGA26*, *PMR1*, *PMT1*, *PMT4*, *PMT6*, *RBR1*, *UTR2*, and *YNK1*, were found to be upregulated in the dispersed cell population. To further characterize the dispersed cells, we developed two separate biofilm assays using two independent C. albicans strains in which the hyphal wall protein *HWP1* gene and yeast wall protein *YWP1* gene were fused with the red fluorescent protein gene, *RFP*. These reporter strains showed that ~33% of dispersed yeast cells expressed hypha-specific *HWP1*, whereas *YWP1* was expressed in ~64% of dispersed yeast cells. This discrepancy, seen in a third of dispersed cells, between their growth form and their expression of a cell type-specific cell wall gene raised the issue of whether these cells can be classified as yeast.

### *PES1*, essential in C. albicans yeast, was required for dispersal cell formation in cells induced or repressed for *NRG1*.

Since the transcriptional profile of dispersed cells most closely resembled that of hyphal biofilm cells, while their morphological appearance corresponded to yeast, we examined dispersed cells by a genetic criterion of cell type: essentiality of the inducer of lateral yeast growth, *PES1*. *PES1*, whose homologues are essential in all eukaryotes, is required for C. albicans yeast cell growth, but hyphae tolerate its depletion from repressible promoters (7). Overexpression of *PES1* in biofilms induces increased lateral yeast production and dispersal from hyphal layers of the biofilm, while its depletion represses release of dispersal cells (10). Overexpression of *NRG1*, a negative regulator of filamentation, also results in significantly increased production of dispersed cells (12) from a predominantly hyphal biofilm. *PES1* expression was elevated >1.7-fold in dispersed cells compared to biofilm cells. Increased (1.4-fold) *NRG1* expression also trended toward significance (*P* value, 0.03; p-adj, 0.08) in dispersed versus the biofilm cells.

**FIG 2:**
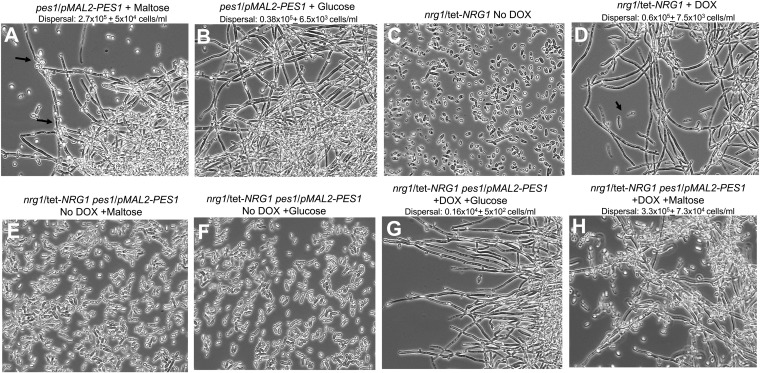
Genetic interaction study of *PES1* with *NRG1* under biofilm conditions. Biofilms were developed overnight in YNB broth in the presence of glucose or maltose as the carbon source and in the presence or absence of DOX. The following strains were used: *pes1*/*pMAL2-PES1*, *nrg1*/tet-*NRG1*, and *nrg1*/tet-*NRG1 pes1*/*pMAL2-PES1*. Biofilms were visualized by light microscopy. Arrows in panel B and D point to the lateral yeast cells, and the frequency of dispersal is indicated in their respective panels.

We tested the requirement for *PES1* in dispersal cells in which *NRG1* expression was exogenously manipulated in a static biofilm model. For this purpose, a *pes1*/*pMAL2-PES1 NRG1*/*tetO-NRG1* strain was constructed and compared with strains containing similar mutations in *PES1* or *NRG1* alone. During induction of *PES1* in the presence of maltose, the *pes1*/*pMAL2-PES1* strain produced a biofilm with abundant lateral yeast cells (dispersal, ~2.7 × 10^5^ cells/ml) (Fig. 2A), while in glucose (during depletion of *PES1*), levels of lateral yeast cells were significantly decreased (dispersal, ~0.38 × 10^5^ cells/ml) (Fig. 2B). Upregulation of the hyphal-growth-repressing *NRG1* gene abrogated biofilm formation (Fig. 2C), while abundant hyphae and a robust biofilm were produced during *NRG1* repression (yeast nitrogen base [YNB] plus doxycycline [DOX]) (Fig. 2D). *PES1* up- or downregulation when *NRG1* was overexpressed in the absence of doxycycline did not reverse the biofilm defect; cells overexpressing *NRG1* remained in the yeast form, rendering them incapable of biofilm formation, regardless of the level of *PES1* expression (Fig. 2E and F).

In contrast, lateral yeast production was significantly impacted by *PES1* induction or repression in *NRG1-*repressed hyphal biofilms. Downregulation of *PES1* abrogated lateral yeast formation and biofilm dispersal (Fig. 2G), while induction of *PES1* expression in *NRG1*-downregulated biofilm cells resulted in prolific lateral yeast production and in a 3-fold increase in biofilm dispersal (Fig. 2H) compared to the *NRG1*-downregulated biofilm alone (Fig. 2D). Hence, dispersal cell formation is strongly impacted by exogenous up- or downregulation of expression of a gene essential in yeast cells, *PES1*. These findings indicate that dispersal cells have genetic properties of yeast and that *PES1* acts downstream of *NRG1* for dispersal cell production.

**FIG 3:**
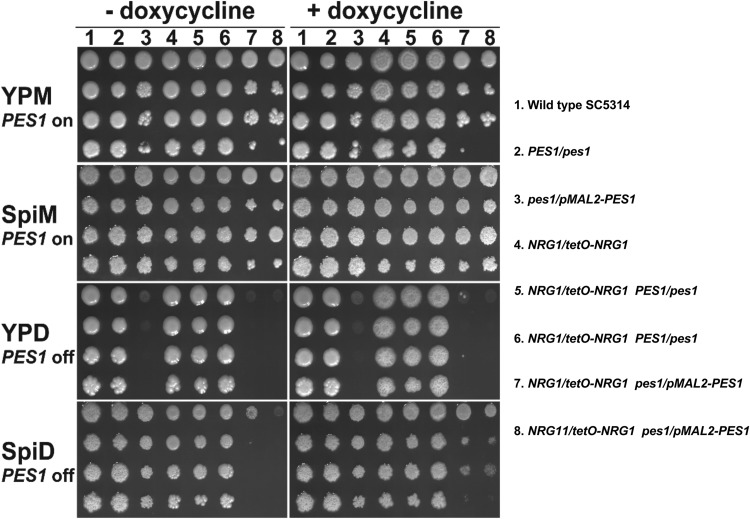
Genetic interaction of *PES1* with *NRG1* on solid media. Serial dilutions of strains were spotted onto YP or Spider medium with 2% glucose or maltose as the carbon source, with or without DOX (5 µg/ml). Lane 1, wild-type SC5314; lane 2, *PES1*/*pes1*, JKC619; lane 3, *pes1*/*pMAL2-PES1*, JKC673; lane 4, *NRG1*/*tetO-NRG1*, SSY50-B; lane 5, *NRG1*/*tetO-NRG1 PES1*/*pes1*, JKC869; lane 6, *NRG1*/*tetO-NRG1 PES1*/*pes1*, JKC870; lane 7, *NRG1*/*tetO-NRG1 pes1*/*pMAL2-PES1*, JKC993; lane 8, *NRG1*/*tetO-NRG1 pes1*/*pMAL2-PES1*, JKC996. Plates were photographed after 3 days of incubation at 37°C (Spider) or 30°C (YP).

In control experiments, we tested relationships of *NRG1* and *PES1* for growth on yeast- or hypha-inducing solid media. Consistent with previous findings, a glucose-repressible *pes1*/*pMAL2-PES1* strain failed to grow at 30°C on yeast extract–peptone–2% dextrose (YPD) medium with 2% glucose and yet grew under hypha-inducing conditions, at 37°C on Spider medium containing glucose as the carbon source (Fig. 3, lane 3). *NRG1*/*tetO-NRG1* strains with wild-type (WT) *PES1* grew on all media, with the growth comprised primarily of yeast during *NRG1* upregulation (− doxycycline), and primarily of hyphal cells during *NRG1* repression (+ doxycycline) as previously established by others [28]) (Fig. 3, lanes 4, 5, and 6). When *PES1* was depleted in *NRG1*-overexpressing cells, growth ceased (−doxycycline, lanes 7 and 8), indicating that those cells were *PES1* dependent. In contrast, when *PES1* was depleted in *NRG1*-repressed cells, those hyphal cells grew robustly (+ doxycycline, lanes 7 and 8). Hence, during *PES1* depletion, growth depended on decreasing genetically determined hyphal repression levels, indirectly supporting the idea that dispersed cells exhibit genetic characteristics of yeast cells.

### Genes differentially regulated exclusively in dispersed cells acted in zinc, iron, and amino acid acquisition.

A total of 335 genes (167 upregulated and 168 downregulated) were found to be differentially expressed in dispersed cells compared to both biofilm and planktonic conditions (listed in sheet 2 in Data Set S2). The predominant group of genes upregulated in dispersed cells represented high-affinity transporters for zinc (*ZRT1* and *ZRT2*) (29) (and a regulator of the two zinc transport genes [*ZAP1*]), lactate (*JEN1* and *JEN2*) (30), and iron (*FET3*, *CFL2*, *CFL4*, and *CFL5*); for amino acid transport (*GAP1*, *GAP2*, *AAP1*, *UGA4*, *CAN1*, *ANT1*, *PTR2*, *OPT1*, and *OPT8*); and for mitochondrion-associated transport (*YHM1*, *YHM2*, *PET9*, *NDE1*, *MIR1*, and *CRC1*) (Fig. 1).

10.1128/mBio.01338-18.3 DATA SET S2  Data set of genes expressed in dispersed cell populations. In this Excel sheet, sheet 1 shows differential expression of central carbon metabolism genes in both dispersed and planktonic cells versus biofilm cells. Here the dispersed cells show a planktonic-yeast-like expression pattern. Sheet 2 shows a set of genes that are upregulated and downregulated only on dispersed cells. These data were extracted after pairwise comparisons of all the sample conditions to each other, picking largely the genes that were upregulated or downregulated exclusively in dispersed cells. Download DATA SET S2, XLSX file, 0.03 MB. Copyright © 2018 Uppuluri et al. 2018 Uppuluri et al. This content is distributed under the terms of the Creative Commons Attribution 4.0 International license.

Genes associated with morphogenesis and biofilm growth had an expression pattern in dispersed cells distinct from those seen with biofilm and planktonic cells (*CSA1*, *YNK1*, *PGA26*, *GNA1*, *RBT1*, *ALS5*, *ALS6*, *IFF4*, and *UTR2*). Of the 168 genes downregulated exclusively in the dispersed cells, 60 had unknown biological functions. While the remaining 108 genes were not readily categorized into functional groups, a number either had functions in mRNA binding or coded for proteins with predicted activity in mRNA splicing via the spliceosome, e.g., *ZSF1*, orf19.285, *EXM2*, orf19.2261, orf19.7139, and *FGR16*. Other downregulated dispersed cell-specific genes were predicted to function in retrograde transport from the endosome/endoplasmic reticulum (ER) to the Golgi compartment, including *VPS41*, *VPS17*, *UFE1*, *MSO1*, *HNM3*, orf19.2333, and orf19.5114. The significance of downregulation of these genes in dispersed cells remains to be determined.

### Telomere-associated (*TLO*) gene family expression showed stark differences between dispersed and biofilm-associated cells.

A set of genes that exhibited significant differences in expression between biofilm cells, planktonic cells, and dispersed lateral yeast cells was the telomeric open reading frame (TLO) gene family. This gene family, whose products are thought to be components of the transcription-regulating mediator complex (31), is comprised of 13 telomere-adjacent genes (*TLO1* to *TLO5* [*TLO1–5*], *TLO7–13*, and *TLO16*) (32, 33) and the nontelomeric family member *TLO34*. In dispersed cells, most *TLO* genes were downregulated at least 4-fold relative to biofilm cells and 2-fold relative to planktonic cells. Therefore, several *TLO* genes (*TLO1*, *TLO2*, *TLO5*, *TLO7*, *TLO8*, *TLO11*, *TLO13*, and *TLO16*) were most highly expressed in biofilm cells, with *TLO2* expressed at a level that was 7-fold higher in biofilms and 2-fold higher in planktonic cells than in dispersed cells.

**FIG 4:**
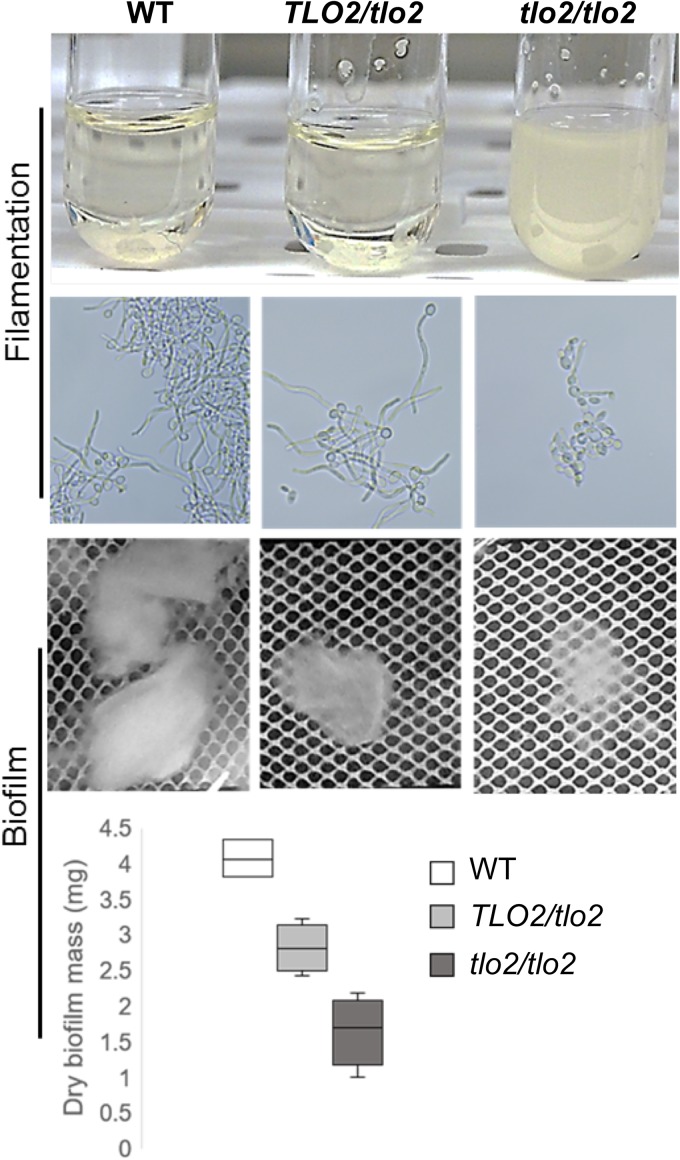
Filamentation and biofilm-forming capabilities of a C. albicans *TLO2* mutant strain. *Candida* strains (wild type, *TLO2* heterozygote, and *tlo2*/*tlo2* homozygote) grown overnight in YPD at 30°C were inoculated into Spider media and incubated at 37°C for 0, 2, 4, and 6 h. Aliquots from each strain were assessed for cell phenotype by microscopy. Biofilms for these strains were also developed under static conditions for 48 h and visualized microscopically, and their dry weight was measured for biomass. The *tlo2*^*−*/*−*^ homozygote showed a drastic reduction in both filamentation and biofilm formation compared to the wild-type strain. The heterozygote displayed normal hyphal development, but the hyphae did not clump together as was seen in the wild-type samples. The *tlo2*/*TLO2* heterozygote also had an intermediate defect on biofilm formation.

We investigated the morphogenesis, biofilm cell, and dispersed cell phenotypes of a mutant lacking *TLO2*, since expression of this gene most distinguished biofilm cells from dispersed and planktonic cells. The *tlo2*^−/−^ strains were found to be defective in filamentation (Fig. 4); cells remained uniformly suspended in liquid Spider medium. This observation was in contrast to the observation of clumps of adherent filaments produced by the wild-type and heterozygous parent strains, which settled to the bottom of the tube. The *tlo2*^−/−^ strain produced yeast cells almost exclusively (Fig. 4). Under conditions of static biofilm induction, *tlo2*^−/−^ showed a significant (~60%) decrease in biofilm growth compared to the wild-type strain (Fig. 4), with the *TLO2*/*tlo2*^−^ strain having an intermediate phenotype (~31% decrease). Biofilms formed by the null mutant were fragile and tended to disintegrate easily, as a consequence of the deficiency in developing scaffolding filaments. Given their defects in biofilm formation, we were not able to assess the dispersal phenotypes of *tlo2*^−/−^ mutants. Further experimentation with, e.g., repressible *TLO2* alleles, whose expression can be downregulated after a biofilm has formed, will be required to define the distinct roles of Tlo2 in biofilm cells versus dispersed cells. Such experiments could resolve the issue of which transcriptional targets of Tlo2 are differentially regulated in hyphal biofilm cells and their dispersed cell daughters.

### Dispersed cells resembled planktonic cells with respect to carbon metabolism.

Under conditions of a continuous flow of fresh medium rich in glucose (1%), biofilm cells expressed much (3-to-9-fold) higher levels of several key genes encoding glycolysis pathway enzymes (*GAL4*, *PGI1*, *PFK1*, *PFK2*, *FBA1*, *PGK1*, *ENO1*, and *CDC19*), relative to planktonic cells grown for 24 h. Dispersed cells released from the same biofilms displayed the opposite pattern of expression, where all glycolysis genes were found to be repressed and core genes required for alternative carbon metabolism were highly upregulated (Data Set S2). This pattern of gene expression in dispersed cells resembled that of planktonic cells after 24 h of growth, wherein elements of the gluconeogenesis pathway—*PCK1* and *FBP1*—exhibited 6-fold and 3-fold increases, respectively, relative to biofilm cells. Genes encoding enzymes of the tricarboxylic acid (TCA) pathway presented the greatest differences, as all were upregulated >10-fold in dispersed cells, as well as planktonic cells, versus biofilm cells (Data Set S2, sheet 1). *ICL1*, which encodes a major enzyme of the glyoxylate pathway, was upregulated 14-fold in dispersed cells and >20-fold in planktonic cells relative to biofilms. Thus, dispersed cells sharply differed from their hyphal mothers. They resembled stationary-phase planktonic cells in their upregulation of alternative carbon metabolism-related genes, despite experiencing a glucose-rich environment.

### Expression patterns shifted in lateral yeast cells prior to their release from the biofilm.

**FIG 5:**
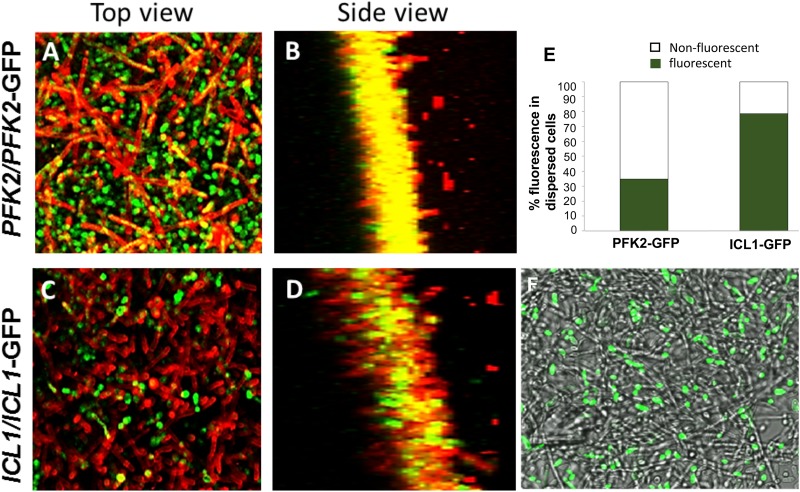
Carbon source utilization signature of biofilm and biofilm dispersal cells. C. albicans cells harboring a GFP-tagged glyoxylate pathway gene, *ICL1* (*ICL1*/*ICL1-GFP*), and a GFP-tagged glycolytic gene (*PFK2*/*PFK2-GFP*) were allowed to form biofilms individually, for 48 h. The biofilm cells were stained with ConA, which stains the cell walls of the fungal cell red, and z-stacks were collected at two wavelengths, 488 nm (GFP) and 594 nm (ConA). (A to D) Top and side-scatter images reveal that the topmost hyphal layers of the biofilm expressed Pfk2 (A and B, respectively) rather than Icl1 (C and D, respectively). (E) In contrast, the cells dispersed from the biofilms expressed Icl1 at a significantly higher frequency than Pfk2. (F) Those results can be visualized by overlaying a bright-field image and GFP fluorescence image of the biofilm, with the result showing brightly fluorescent yeast cells on top of a mat of nonfluorescent *ICL1-GFP* biofilms.

To visualize the disparity of carbon metabolism gene expression between biofilm cells and dispersed cells at the protein level, we used strains expressing green fluorescent protein gene (*GFP*)-tagged *PFK2* (a glycolysis gene) or *ICL1* (a glyoxylate cycle gene). Biofilms were developed under the same growth conditions as those of cells analyzed by RNA-seq. Following a 24-h growth period, biofilms were stained using Alexa 594-conjugated concanavalin A (ConA; a lectin that binds to mannans of the fungal cell wall) and visualized by confocal microscopy. A top-down view of *PFK2*-GFP biofilms displayed a high intensity of green fluorescence (with yellow streaks in hyphae due to overlapped red ConA signal), suggesting that biofilm cells were undergoing glycolysis. Merging signals of the side view of the biofilm showed overlapping signals, indicating a potential for glycolytic activity in hyphae stained by ConA (Fig. 5A and B; see also slide 1 of Fig. S1 for alternative images). In contrast, only a small proportion of cells in the *ICL1*-GFP biofilms displayed green fluorescence (Fig. 5C and D), suggesting that the glyoxylate cycle was largely inactive in the biofilms.

10.1128/mBio.01338-18.1 FIG S1  Figures related to Fig. 2 in the manuscript. Slide 1 shows that the topmost layers of the flow biofilm expressed green fluorescent Pfk2. Slide 2 shows that the cells attached to the silicone substrate (innermost portion of the flow biofilm) expressed green fluorescent Icl1. Download FIG S1, TIF file, 1 MB. Copyright © 2018 Uppuluri et al. 2018 Uppuluri et al. This content is distributed under the terms of the Creative Commons Attribution 4.0 International license.

Interestingly, dispersed cells expressed *ICL1* at a significantly higher rate than *PFK2* (Fig. 5E). The dispersed cells from the *PFK2-GFP* biofilms displayed an overall dim fluorescence, and only about 35% of cells showed a clear fluorescent signal. In contrast, ~78% of the dispersed cells from the *ICL1-GFP* biofilms showed bright green fluorescence. A merged bright-field–GFP fluorescence image of the *ICL1-GFP* biofilm surface clearly demonstrated that GFP fluorescence was evident only in lateral yeast cells, i.e., those poised for release from the hyphal surface, and not in the hyphal mat (Fig. 5F).

**FIG 6:**
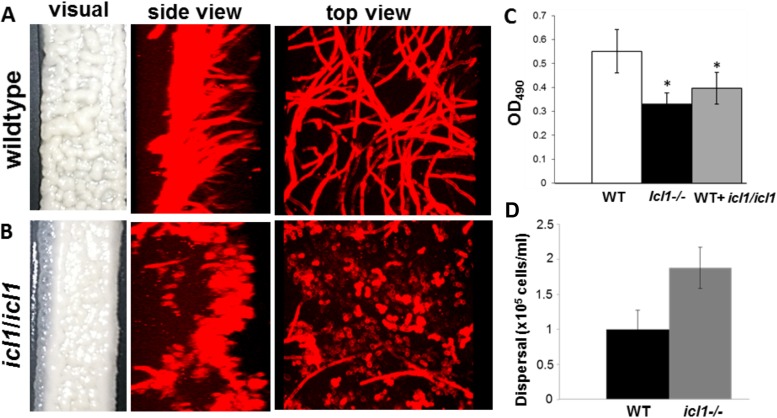
Impact of *ICL1* loss on biofilm formation and dispersal. Biofilms of C. albicans wild-type and *icl1*/*icl1* strains were allowed to form for 48 h. (A to C) Macroscopic and microscopic observations (biofilms stained with ConA and imaged by confocal laser scanning microscopy [CLSM]) revealed that the biofilms formed by the mutant (B) were less robust than those formed by the wild-type strain (A), which was confirmed by XTT metabolic assay (C). (D) Nonetheless, the level of dispersal from *icl1* mutant biofilms was higher than that from the wild-type biofilms.

We wanted to examine whether Icl1 contributes to dispersal. However, a C. albicans *icl1*^−/−^ mutant appeared to form a significantly less robust biofilm by macroscopic inspection. Confocal microscopy confirmed that *ici1*^−/−^ cells had a defect in filamentation under biofilm conditions (Fig. 6A and B); however, no defects were noted during planktonic growth. Yeast cell dispersal from *icl1*^−/−^ biofilms was increased 2-fold over that from the wild-type biofilms, indicating that induction of dispersal, but not biofilm formation, proceeds independently of this protein (Fig. 6D). Although *ICL1* contributes to the metabolic activity of biofilm growth, its detection was perhaps below the limit of detection under the experimental conditions used for RNA-seq. Given this paradox, we detached the biofilm in its entirety (by using forceps), inverted it, and imaged its underside as well as the thin basal layer of cells still attached to the silicone material. Images revealed *ICL1*-*GFP* signal in several patches within the innermost layers of the biofilm, indicating expression of Icl1 in this environment (Fig. S1, slide 2). All the more striking, *ICL1* was expressed in lateral yeast cells at the surface of the biofilm, where ambient nutrients are abundant. Taken together, these results suggest that a biofilm harbors subpopulations of cells which have their own unique gene expression pattern. This was shown by *ICL1* expression within the deeper layers of the biofilm, where the cells experience substantial nutrient limitation. This finding implies that lateral yeast cells undergo metabolic reprogramming before release from the biofilm.

### Control of biofilm dispersal inhibited C. albicans dissemination in jugular vein-catheterized mice.

To investigate whether modulation of dispersal from biofilms *in vivo* controls biofilm-mediated invasive disease, catheters were inoculated intraluminally with a *pes1*/*tetO-PES1* strain in which the expression level of the major regulator of lateral yeast production, *PES1*, was controlled by addition or removal of DOX in the environment (7).

**FIG 7:**
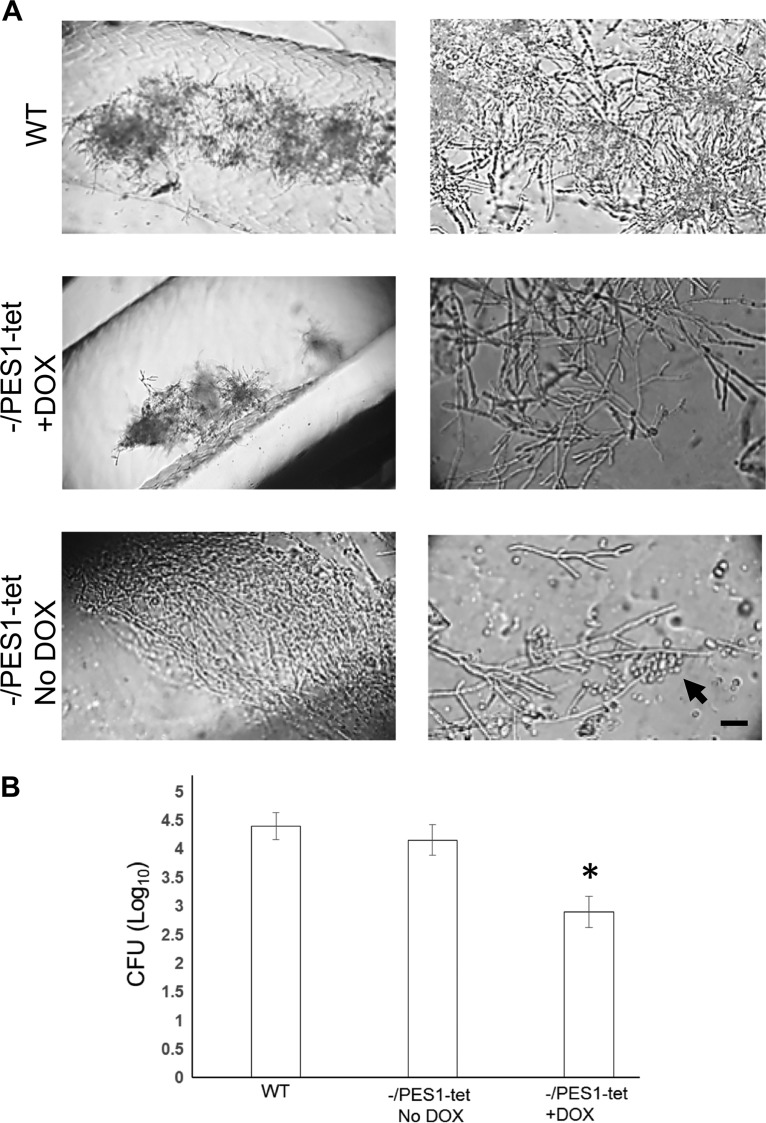
Biofilm formation and dispersal in jugular vein-catheterized mice. (A) Germinating C. albicans tetracycline-regulatable *PES1* strain *pes1*/*PES1-tet* grown in YNB medium with and without doxycycline was instilled in the lumen of the catheters (full catheter volume) at a concentration of 5 × 10^6^ cells/ml. Biofilms were allowed to develop for 3 days, after which the catheters were harvested, cut laterally, and examined under a phase-contrast microscope. While all 3 strains developed biofilms in the catheters in the presence or absence of DOX, the Pes1-tet hyphae displayed increased lateral yeast production in the absence of DOX (arrow). Bar, 20 µm. (B) The extent of biofilm-mediated dissemination in mice harboring the catheters containing the two strains was also determined by measuring CFU levels in the kidney. In the presence of DOX, dissemination and kidney colonization were reduced significantly (15-fold). *, *P* < 0.01 (compared to the mice with catheters infected with the wild-type strain or with Pes1-tet without DOX).

After 3 days, catheters were recovered and the distal 2 cm of the catheter was cut into pieces to measure the level of metabolic activity by using the XTT [2,3-bis-(2-methoxy-4-nitro-5-sulfophenyl)-2H-tetrazolium-5-carboxanilide salt] assay. Regardless of the presence or absence of DOX in the catheter, the *pes1*/*tetO-PES1* biofilms and the WT biofilms displayed the same XTT readout, indicating equivalent results with respect to biofilm formation on catheters (data not shown). Microscopy of the biofilms revealed that all catheters harbored biofilms containing hyphal filaments; catheters in which *PES1* was overexpressed (in the absence of DOX) produced a high number of lateral yeast cells from the hyphal filaments (Fig. 7A). The levels of dissemination and kidney colonization were comparable between these catheters and wild-type-biofilm-containing catheters. In contrast, biofilms in catheters treated with DOX to repress *PES1* lacked lateral yeast cells, and the kidneys of these mice showed a 1.5 log decrease in C. albicans dissemination and kidney colonization (Fig. 7).

## DISCUSSION

Biofilm formation is the predominant mode of growth for most microorganisms in natural and clinical environments (34). For many pathogenic organisms, biofilm dispersal plays a critical role in the transmission of cells between hosts and in the propagation and spread of infection within a single host (35). For example, Streptococcus mutans can detach from dental biofilms in a mother’s mouth and be transmitted to an infant by direct or indirect contact (36). Examples of intrahost spread encompass hospital-acquired pneumonia caused by bacteria detached from biofilms in a patient’s endotracheal tube (37); an extreme case of hematogenous spread of C. albicans into the eye during urinary sepsis arising from an infected ureteric stent (38); and dissemination of C. albicans from biofilms on intravenous catheters, as we modeled in this study. Hence, understanding the characteristics of dispersed cells is important to understand how C. albicans adapts once released from the biofilm.

We have previously reported that dispersed cells exhibit a number of characteristics associated with pathogenesis compared to planktonically grown yeast cells (10), indicating measurable differences between the molecular signatures of these two different populations of yeast cells. To address that issue, in this study we performed global comparative transcriptional profiling on dispersed cells. The findings from the RNA sequencing-based analysis of gene expression patterns defined characteristics that largely overlapped those of their biofilm mothers and contrasted with those of planktonic yeast. However, these cells exhibited striking differences from the biofilm cells with which they shared similar growth conditions with respect to the genes required for attachment and invasion of a host. In addition, RNA sequencing data replicated our previous studies comparing biofilm cells and planktonic cells (15, 16). Collectively, our findings provide evidence for the presence of a developmental program that is activated during the cell dispersal process, as detailed below.

Given gene expression patterns that were obviously different from those of planktonic yeast, we wished to define the cell type of dispersal cells beyond their morphological appearance. To do this, we examined the genetic relationship between *PES1* and *NRG1* in these cells, because *PES1* is known to be essential in C. albicans yeast. We previously reported that expression levels of *PES1* and *NRG1* regulate the rate of C. albicans biofilm dispersal cell production (10, 12). We have now found that in cells locked in the yeast form during *NRG1* overexpression, *PES1* depletion became synthetically lethal. In contrast, hyphal induction through extracellular signals or through *NRG1* repression permitted biofilm growth during *PES1* depletion. *PES1* overexpression suppressed the filament-locked phenotype of *NRG1*-depleted biofilms to generate a profusion of lateral yeast cells, suggesting that *PES1* acts downstream of *NRG1* in regulating this developmental step. Further experiments will be needed to understand the molecular basis of these observations.

We note that the yeast-specific *YWP1* gene was lowest in expression in the planktonic yeast cells. In order to maintain the nutritional conditions of biofilm cells and planktonic cells to be as similar as possible, planktonic cells were grown in YNB medium at 37°C, which may produce a mixed population of yeast and pseudohyphal cells that as a result do not express YWP1 robustly, thereby resulting in some atypical expression patterns for yeast.

Differential gene expression studies in our flow biofilms compared to age-matched planktonic cells revealed similarities in more than 180 core genes known to have roles in biofilm development (15, 16, 39) (see Data Set S1 in the supplemental material). Compared to planktonic cells, several genes related to adhesion and filamentation were upregulated in dispersed cells to an extent comparable to that seen in biofilm cells. This provided a molecular basis for our previous observation that dispersed cells are virulent entities (9, 10). Biofilm cells secrete products of a number of *SAP* genes at high levels as biofilm-specific proteases (candidapepsins) (26), and several of these genes (*SAP3*, *SAP6*, *SAP8*, and *SAP9*) were also found in the present study to be highly expressed in biofilm dispersal cells. *SAP* genes in C. albicans have several virulence functions, including host tissue adhesion, invasion and damage, or destruction of cells and molecules of the host immune system to resist antimicrobial attack (40). Thus, with the continued expression of *SAP* genes, the dispersal cells remain “armed” to invade the host.

In variable environments, microbes may enhance their fitness by predicting and preparing for a coming change (41). As an example, the enteric bacterium Escherichia coli elicits a transcriptional response for hypoxia when shifted from growth at 30°C to 37°C (42), as the increased temperature may indicate its arrival in the gut, where oxygen tends to be scarce. Similarly, C. albicans responds to higher pH by expressing genes involved in iron and zinc uptake via the alkaline-induced transcription factor Rim101 (43). Since the two metals are less soluble at high pH, the fungus predicts metal starvation based on its current growth environment. We posit, therefore, that dispersed C. albicans cells similarly express genes required for adhesion and tissue invasion in an anticipatory manner, based not on external cues but on a developmental program. Genes controlling zinc (*ZAP1*, *ZRT1*, and *ZRT2*), iron, and amino acid acquisition were found to be exclusively upregulated in the dispersed cells, though they were exposed to the same ample abundance of nutrients as their biofilm-associated mothers.

Zinc-dependent signaling has been shown previously to affect the ratio of hyphae to yeast in a biofilm; reduced expression of *ZAP1*—a regulator of zinc transport genes *ZRT1* and *ZRT2*—in a biofilm leads to yeast accumulation (44). Perhaps lower *ZAP1* expression levels in biofilm cells trigger yeast accumulation and dispersal. Upregulation of *ZRT1* and *ZRT2* in dispersed cells might follow *ZAP1* expression elevation in this population.

Strikingly, dispersed cells strongly induced expression of genes encoding elements of the gluconeogenesis and glyoxylate pathways, required during carbon source starvation, while in the same rich medium, their biofilm mothers expressed a metabolic gene profile reflective of glycolysis. We conjecture that this discordant relationship between nutrient availability and expression of genes required during starvation such as those encoding transporters and glyoxylate cycle components reflects an anticipatory developmental step during production of cells primed to infect new sites in the host with low availability of glucose and other crucial nutrients. In summary, it is tempting to speculate that increased expression of adhesin genes, hypha-specific genes, and secretory aspartyl protease genes as well as of gluconeogenesis-associated and glyoxylate cycle-associated genes in biofilm dispersal cells or planktonic cells suggests that in biofilm-released cells, gene products required during or after tissue invasion are induced before the actual invasion process is initiated.

Unlike the yeast-to-hypha transition, which requires a specific external trigger (such as heat, serum, specific nutrients, alkaline pH, hypoxia, etc.), the hypha-to-lateral-yeast developmental process occurs constitutively. In nutritionally rich or poor media (7, 45, 46), in host tissues (8), or in biofilms formed in catheters (10), C. albicans hyphae consistently produce lateral yeast cells. Hence, this process appears to reflect an intrinsic developmental program. *TLO* genes, which are broad transcriptional regulators, are differentially upregulated in biofilm cells. Whether the mechanism underlying differential gene expression in a biofilm mother cell and its dispersed daughter consists of selective mRNA transport between the cytoplasm of these cells or, e.g., of reprogramming of gene expression after mitosis of the dispersed cell remains to be determined in future work.

In conclusion, our findings confirm the importance of *PES1* for C. albicans yeast growth and its significant impact on lateral yeast production and biofilm dispersal. Regulation of *PES1* could have important implications in clinical settings, where biofilms formed on catheters have direct access to the bloodstream. This was seen in our *in vivo* studies using the jugular vein catheter mouse model where overexpression of *PES1* in biofilms growing in the lumen of the catheters resulted in enhanced dispersal and disseminated infection, while repression of *PES1* resulted in abrogation of biofilm dispersal and a 15-fold decrease in *Candida* infection of distal organs. These findings may lay the foundation for discovery of novel inhibitors that interfere with the regulatory signals controlling dispersal from biofilms. A better understanding of the molecular mechanisms of biofilm dispersal might pave the way for control of biofilm-mediated disseminated diseases in humans.

## MATERIALS AND METHODS

### Strains and culture conditions.

Stock cultures of all strains were stored in 15% glycerol at −80°C. Strains were routinely grown under yeast conditions (media at 30°C) in YPG (1% yeast extract, 2% Bacto peptone, 2% glucose) or under filament-inducing conditions using RPMI medium (Sigma, St. Louis, MO) with MOPS (morpholinepropanesulfonic acid) buffer or Spider medium. The strains used in this study, their origin, and their construction are briefly listed in Table 1.

**TABLE 1:** List of strains used in this study

Strain	Genotype/construction	Reference or source
SC5314	Wild type	54
CLM3-2	*ura3*::*λ imm434*/*ura3*::*λ imm434*, pPFK2-GFP	55
CJB-3	*ura3*::*λ imm434*/*ura3*::*λ imm434*, pICL1-GFP	55
RM1000	*ura3*::*λ imm434*/*ura3*::*λ imm434*, *his1*::*hisG*/*his1*::*hisG*	56
CLM18-1	*ura3*::*λ imm434*/*ura3*::*λ imm434*, *his1*::*hisG*/*his1*::*hisG*, *icl1*::*HIS1*/*icl1*::*URA3*	55
JKC619	*pes1*::*FLP-NAT1*/*PES1* (parent SC5314)	7
JKC673	*pes1*::*FRT*/*FLP-NAT1-pMAL2-PES1* (parent JKC619)	7
SSY50B	*NRG1*/*tetO-NRG1-URA3 ade2*::*hisG*/*ade2*::*hisG ura3*::*imm434*/*ura3*::*imm434 ENO1*/*eno1*::*ENO1-tetR-ScHAP4AD-3*× *HA-ADE2*	57
JKC869	*pes1*::*FLP-NAT1*/*PES1 NRG1*/*tetO-NRG1-URA3 ade2*::*hisG*/*ade2*::*hisG ura3*::*imm434*/*ura3*::*imm434 ENO1*/ *eno1*::*ENO1-tetR-ScHAP4AD-3×HA-ADE2*; transformant 3; SSY50B was transformed with pJK890 (deletion plasmid to replace *PES1* with *FLP-NAT1* as previously described [7]); parent strain, SSY50-B	This work
JKC870	*pes1*::*FLP-NAT1*/*PES1 NRG1*/*tetO-NRG1-URA3 ade2*::*hisG*/*ade2*::*hisG ura3*::*imm434*/*ura3*::*imm434 ENO1*/ *eno1*::*ENO1-tetR-ScHAP4AD-3×HA-ADE2*; transformant 4; SSY50B was transformed with pJK890 (deletion plasmid to replace *PES1* with *FLP-NAT1* as previously described [7]); parent strain, SSY50-B	This work
JKC993	*pes1*::*FRT*/*FLP-NAT1-pMAL2-PES1 NRG1*/*tetO-NRG1-URA3 ade2*::*hisG*/*ade2*::*hisG ura3*::*imm434*/*ura3*::*imm434 ENO1*/ *eno1*::*ENO1-tetR-ScHAP4AD-3×HA-ADE2*; *FLP-NAT1* was excised from JKC869 by inducing flippase, and the resulting strain was transformed with pJK896 (promoter replacement plasmid to replace *PES1* promoter with *MAL2* promoter as previously described [7]); parent strain, JKC869	This work
JKC996	*pes1*::*FRT*/*FLP-NAT1-pMAL2-PES1 NRG1*/*tetO-NRG1-URA3 ade2*::*hisG*/*ade2*::*hisG ura3*::*imm434*/*ura3*::*imm434 ENO1*/ *eno1*::*ENO1-tetR-ScHAP4AD-3×HA-ADE2*; *FLP-NAT1* was excised from JKC869 by inducing flippase, and the resulting strain was transformed with pJK896 (promoter replacement plasmid to replace PES1 promoter with *MAL2* promoter as previously described [7]); parent strain, JKC870	This work
*TLOβ2*/*tloβ2*	Strain 12822 (haploid strain from Yue Wang, Singapore) was transformed with the PCR product of pGEM-URA3 (56); transformants were selected for on SDC-ura and confirmed by PCR for pGFP-NAT1 (58); ransformants were selected for on YPAD plus neurseothricin and confirmed; PCR with TLO2 primers confirmed that no wild-type copy remained	This work

### Cell collection and RNA extraction.

Biofilms were developed in a simple flow biofilm model as described previously (13, 14). Biofilms were allowed to grow for 24 h in YNB–1% glucose media. The biofilms were collected from the SE strip (Cardiovascular Instruments Corp., Wakefield, MA) and flash frozen in liquid nitrogen followed by short-term storage at −80°C. Prior to harvesting, cells released from the biofilm in the flowthrough media were collected at the completion of the 24-h growth period. These cells were microscopically confirmed to be yeast cells. As biofilms were previously shown to disperse at the rate of ~5 × 10^5^ cells/ml in YNB medium (10), to obtain enough cells for RNA extraction, we collected the flowthrough several times from replicate biofilms (3 ml at a time in tubes placed on ice). The cells were pelleted by cold ultracentrifugation, and the total time from collection of the dispersed cells to freezing of the pellet on dry ice at −80°C was 8 min. For some experiments, dispersed cells were enumerated by hemocytometer and by colony counts on solid medium (YPD-agar plates). Planktonic cells growing for 24 h in flasks containing YNB–1% glucose at 37°C were centrifuged, and the pellets were stored at −80°C. RNA was extracted from all frozen pellets using a RiboPure yeast kit (22, 44).

### Static biofilm formation.

Biofilms were grown under static conditions in microtiter plates and visualized by bright-field or confocal scanning laser microscopy, as previously described (47), with minor modifications. Briefly, 1 ml of C. albicans cells (1 × 10^6^ cells/ml) was added to the wells of a 24-well microtiter plate and incubated overnight in YNB with or without DOX and in glucose or maltose as the carbon source, and then the biofilms formed were measured by an XTT assay as previously described (47). For enumeration of dispersed cells, static biofilm supernatants (and one wash) were collected and cells counted with a hemocytometer. Biofilms were also grown on catheter material and quantified by dry weight measurements, as previously published (39, 47, 48). Statistical significance data (*P* values) were calculated with a Student’s *t* test.

### Comparative transcriptomic analysis by RNA sequencing.

RNA was extracted from a total of six samples: two replicates each of the planktonic cells, the biofilm cells, and the biofilm dispersal cells. RNA sequencing and bioinformatic analysis were performed at the Genome Sequencing Facility of Greehey Children’s Cancer Research Institute at the University of Texas Health Science Center at San Antonio, Briefly, 5 µg total RNA was used for mRNA isolation with Dynabeads OligodT (Invitrogen, Carlsbad, CA), and then about 30 to 50 ng isolated mRNA was used for mRNA-seq library preparation by following the sample preparation guide provided with a BIOO Scientific NEXTflex Directional RNA-seq kit (dUTP-based). The first step in the workflow involves purifying the poly(A)-containing mRNA molecules using Dynabeads poly(T) oligonucleotide-attached magnetic beads. Following purification, the mRNA is fragmented into small pieces using divalent cations and elevated temperature (95°C for 10 min). The cleaved RNA fragments are copied into first-strand cDNA using reverse transcriptase and random primers. This is followed by second-strand cDNA synthesis using DNA polymerase I and RNase H. These cDNA fragments then go through an end repair process, the addition of a single “A” base, and then ligation of the adapters. The products are then purified and enriched by PCR to create the final RNA-seq library. Directionality is retained by adding dUTP during the second-strand synthesis step and by subsequent cleavage of the uridine-containing strand using uracil-DNA glycosylase. After the RNA-seq libraries were subjected to the quantification process and pooled for cBot amplification, subsequent 100-bp-paired-end sequencing was run with an Illumina HiSeq 2000 platform (San Diego, CA). After the sequencing run, demultiplexing with CASAVA was employed to generate the fastq file for each sample. All sequencing reads were filtered, trimmed, and aligned with a C. albicans reference genome using TopHat2 (49) default settings, and the Bam files from alignment were processed using HTSeq-count (50) to obtain the counts per gene in all samples. A statistical analysis of differential gene expression was performed using the DESeq package from Bioconductor (51), and a gene was considered significantly altered if the false-discovery rate for differential expression was ≤0.05. The processes associated with differentially expressed genes were identified using Gene Ontology Slim Mapper (52).

### Epistasis study on solid media.

Cells were grown on yeast extract–peptone–2% maltose (YPM) plates containing 5 µg/ml doxycycline (DOX) for 48 h at 30°C. Following two washes with 0.9% NaCl, 5-fold cell dilutions (beginning at an optical density at 600 nm [OD_600_] of 0.5) were spotted with a calibrated replicator (V&P Scientific, San Diego, CA) onto YPM, yeast extract–2% dextrose (YPD), or Spider medium without mannitol with 2% maltose (SpiM) or with 2% dextrose (SpiD). Plates were incubated for 3 days at 30°C (YP) or 37°C (Spi). Plates contained DOX (5 µg/ml) or vehicle.

### Jugular vein catheter mouse model of infection.

A C. albicans mouse catheter biofilm model was used for *in vivo* experiments as previously described (53) with minor modifications. These *in vivo* experiments were approved by the Los Angeles Biomedical Research Institute, Harbor-UCLA IACUC. Briefly, we used catheterized 8-week-old C57BL/6 male mice, purchased from laboratories of Charles River, Inc. (Wilmington, MA), where the surgery was performed. The surgery involves insertion of a Silastic catheter into the jugular vein of the mice. Patency is tested, and the catheter is filled with heparin lock solution and sealed with a plug. Following receipt of the jugular vein-catheterized mice, the catheters were instilled with 25 µl of a C. albicans inoculum of 5 × 10^6^ cells/ml (the entire catheter volume) using a 23-gauge blunt-ended needle after removal of the plug and the lock solution (the plug was put back in place after inoculation).

The wild-type and *pes1*/*PES1-tet* strains were allowed to germinate for 90 min in the absence of DOX in YNB medium. Mice were divided into the following 3 groups with 5 mice in each group: (i) mice with catheters infected with the wild-type strain in YNB, (ii) mice with catheters infected with the *pes1*/*PES1-tet* strain in YNB, and (iii) mice with catheters infected with the pes1/*PES1-tet* strain in YNB containing 25 µM DOX. Cells were allowed to adhere in the catheter lumen for 3 days, after which the mice were euthanized and catheters aseptically removed. The catheters were cut laterally and imaged under a phase-contrast microscope to visualize the morphology of the cells growing within the catheters of the individual groups. The distal 2 cm of the catheters was cut first laterally and then into small pieces and introduced into a 96-well plate containing XTT solution for 90 min, to measure the metabolic activity of cells in the biofilm. Additionally, kidneys were harvested, weighed, homogenized, and plated on YPD for CFU enumeration. Differences in organ fungal burden between the 3 groups are presented as log CFU per gram tissue, and results from a two-tailed *t* test with a *P* value of <0.05 were considered significant.
