# Supplementary figures and images for: Candida albicans Dispersed Cells Are Developmentally Distinct from Biofilm and Planktonic Cells
Source: mBio. 2018 Aug 21;9(4):e01338-18. doi: 10.1128/mBio.01338-18 (PMC6106089; doi:10.1128/mBio.01338-18)

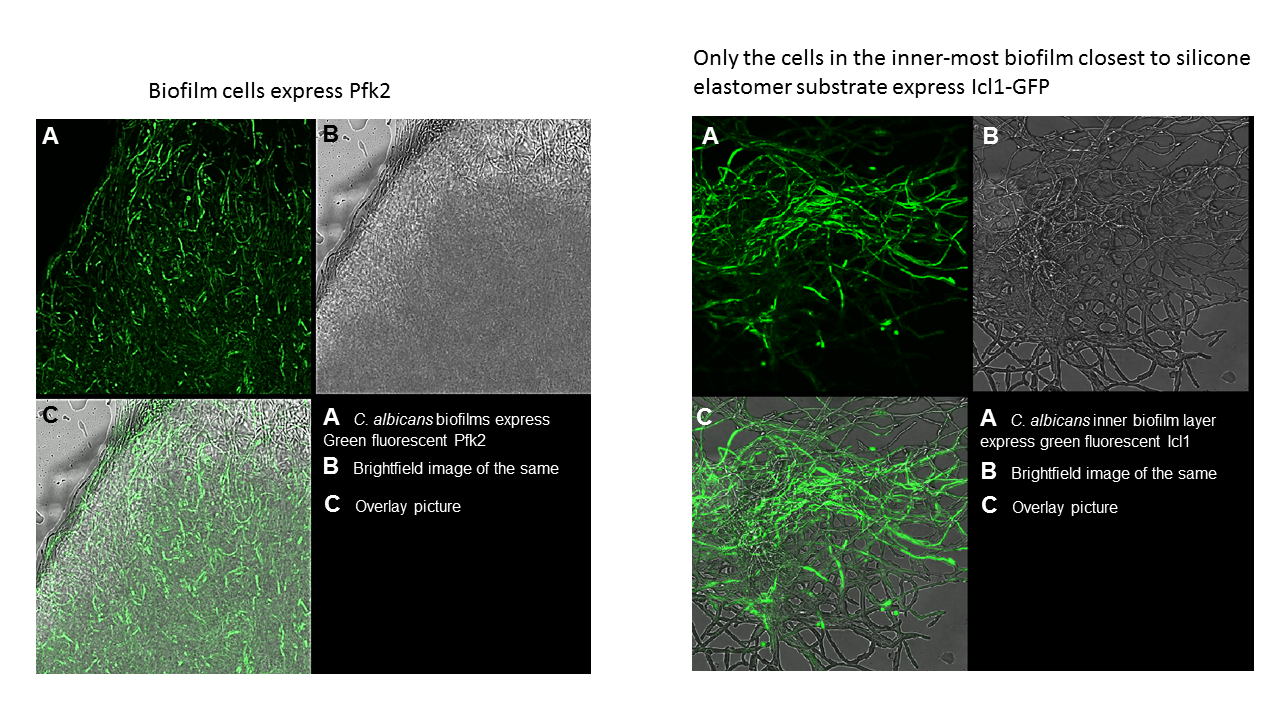

Supplement: FIG S1 [file mbo004184029sf1.tif]
